# Functional Investigation of Plant Growth Promoting Rhizobacterial Communities in Sugarcane

**DOI:** 10.3389/fmicb.2021.783925

**Published:** 2022-01-04

**Authors:** Mingjia Li, Ran Liu, Yanjun Li, Cunhu Wang, Wenjing Ma, Lei Zheng, Kefei Zhang, Xing Fu, Xinxin Li, Yachun Su, Guoqiang Huang, Yongjia Zhong, Hong Liao

**Affiliations:** ^1^Root Biology Center, Fujian Agriculture and Forestry University, Fuzhou, China; ^2^National Engineering Research Center of Sugarcane, Fujian Agriculture and Forestry University, Fuzhou, China

**Keywords:** sugarcane, root-associated microbes, beneficial function, nitrogen, growth promotion

## Abstract

Plant microbiota are of great importance for host nutrition and health. As a C_4_ plant species with a high carbon fixation capacity, sugarcane also associates with beneficial microbes, though mechanisms underlying sugarcane root-associated community development remain unclear. Here, we identify microbes that are specifically enriched around sugarcane roots and report results of functional testing of potentially beneficial microbes propagating with sugarcane plants. First, we analyzed recruitment of microbes through analysis of 16S rDNA enrichment in greenhouse cultured sugarcane seedlings growing in field soil. Then, plant-associated microbes were isolated and assayed for beneficial activity, first in greenhouse experiments, followed by field trials for selected microbial strains. The promising beneficial microbe SRB-109, which quickly colonized both roots and shoots of sugarcane plants, significantly promoted sugarcane growth in field trials, nitrogen and potassium acquisition increasing by 35.68 and 28.35%, respectively. Taken together, this report demonstrates successful identification and utilization of beneficial plant-associated microbes in sugarcane production. Further development might facilitate incorporation of such growth-promoting microbial applications in large-scale sugarcane production, which may not only increase yields but also reduce fertilizer costs and runoff.

## Introduction

Plant-associated microbes colonize organs throughout host plants, with distinctive microbial communities forming in the different niches plants present, and the collection of organisms forming a holobiont (Tringe et al., 2005; Hassani et al., 2018). Within this intimate set of associations, plants provide microbes with photosynthates in exchange for a variety of beneficial features from microbes that confer improvements in host fitness across diverse environments (Dong et al., 2018). The composition of host associated microbial communities may act as an important determinant of plant health and yield through impacts on nitrogen fixation, phosphorus solubilization, 3-indoleacetic acid (IAA) production, root growth, and nutrient acquisition efficiency (Beckers et al., 2017; Wang et al., 2021). Recruitment and enrichment of the beneficial members of the plant microbial community are in turn determined by the available pool of soil microbiota, the host plant genotypes, and host plant nutrient status (Mendes et al., 2011; Zhong et al., 2019).

Sugarcane accounts for 90% of the sugar production in China (Li and Yang, 2015), and the byproducts can also be used for biomass energy production and animal husbandry (de Almeida et al., 2018; Vieira et al., 2020). Sugarcane is also a C_4_ crop with high photosynthetic efficiency and rapid growth, which requires large amounts of mineral nutrients, especially nitrogen, to support growth and coordinate nutrient homeostasis with carbon fixation capacity (Gopalasundaram et al., 2012; Carvalho et al., 2014; de Oliveira et al., 2016). This has led to excessive nitrogen applications in sugarcane production (Boddey et al., 2003). A tradeoff of excessive nitrogen fertilization is serious environment impacts, including soil acidification, water eutrophication, and air pollution (Guo et al., 2010; Le et al., 2010; Liu et al., 2013). Hence, increasing sugarcane production while simultaneously protecting agroecosystems through more efficient nutrient acquisition and decreased fertilization are interconnected practical objectives for improving sugarcane production (Rosenblueth et al., 2018).

Although sugarcane plants need large amounts of nitrogen during vegetative growth, up to 70% of the total nitrogen required for its growth may be acquired through associated nitrogen fixation (Urquiaga et al., 1992). Multiple years of field trials in Brazil sugarcane production systems have demonstrated that associated nitrogen fixation can yield up to 40 kg/ha/y of nitrogen in these fields (Urquiaga et al., 2012). Hence, nitrogen fertilizer applications for sugarcane production in Brazil are significantly lower than in other countries due to the nitrogen fixation associated with Brazilian sugarcane (Boddey et al., 2003; Urquiaga et al., 2012). To date, nitrogen fixing bacteria isolated from sugarcane include *Beijerinckia* spp., *Azospirillum* spp., *Herbaspirillum* spp., *Gluconacetobacter* spp., *Enterobacter* spp., *Burkholderia* spp., *Klebsiella* spp., and so on (Döbereiner and Day, 1976; Boddey and Döbereiner, 1995; Baldani et al., 1997; Oliveira et al., 2009; Mehnaz, 2013; Carvalho et al., 2014; Lin et al., 2015; Muthukumarasamy et al., 2017; Guo et al., 2020). Beyond nitrogen fixation, plant-associated bacteria may also provide numerous other benefits for host plants (Oliveira et al., 2002; Saravanan et al., 2007; Ahmed and Holmström, 2014; Carvalho et al., 2014).

Over the last two decades, while knowledge of associated nitrogen fixation with sugarcane has greatly improved, most of this work focused on specific nitrogen fixing bacteria and lacked a systematic investigation of sugarcane associated communities (da Silva et al., 2018; Singh et al., 2019). Recently, with the development of high throughput sequencing technology, the structure and activities of sugarcane microbiome components have been gradually revealed (Bertalan et al., 2009; Pedrosa et al., 2011; Sant’Anna et al., 2011; Zhu et al., 2012; de Souza et al., 2016; Yeoh et al., 2016; da Silva et al., 2018; Dong et al., 2018; Schwab et al., 2018). In this study, we decipher the composition of a bacterial community in the root and rhizosphere of sugarcane using 16S rDNA sequencing. Then, potentially beneficial microbes were isolated from sugarcane roots and evaluated for plant growth promotion in both greenhouse pot experiments and a field trial. The results provide significant insights into interactions between sugarcane and root microbiota that may be and further harnessed to utilize beneficial microbes in sugarcane production.

## Materials and Methods

### Sugarcane Growth and Cultivation

In order to investigate microbes specifically recruited from soil and enriched in the roots of commercial sugarcane variety ROC22, seedlings were propagated to the four-leaf stage in MS medium prior to transferring to rooting medium for another 2 weeks (Supplementary Figure 1). Transplanted sugarcane seedlings were then acclimatized for another 2 weeks to growth in liquid plant nutrition solution (Li et al., 2015) prior to transplanting into soils collected from a sugarcane farm managed by the Fujian Agriculture and Forestry University. Before planting, soils were filtered to remove larger residues and mixed well with equal volumes of sterile vermiculite, which was heated at 121°C for 40 min before use. Sugarcane seedlings were planted 1 seedling per pot (10 * 10 * 15 cm) in the soil mix described above. A total of 16 pots of sugarcane seedlings were reared in a growth chamber (day/night: 14 h/10 h, 26°C/24°C) under 37.5 μE/m^2^/s of daylight light intensity for 3 months before harvesting samples. During plant growth, liquid plant nutrient solution was supplied according to plant needs.

### Rhizosphere and Root-Associated Microorganism Sampling

To sample the rhizosphere and root-associated microorganisms, sugarcane plants were removed from pots, with loosely attached soil being removed from roots prior to collecting rhizosphere soil firmly attached to the roots. Rhizosphere soils were collected through washing in 100 mL sterile phosphate buffer saline (PBS) solution and centrifugation at 12,000 rpm for 15 min (Bulgarelli et al., 2012, 2015; Zhong et al., 2019). After removing rhizosphere soil, roots were further treated with sonication for 20 min to remove microbes from the root surface (Lundberg et al., 2012), which were collected in three rinses of sterile water (Supplementary Figure 2). Each biological replicate of rhizosphere soil and sugarane root sample was collected from four independent sugarcane plants. In total, four biological replicates were collected for each rhizosphere soil and root.

### DNA Extraction, Sequencing, and Analysis

Total DNA was extracted from rhizosphere soil and root samples using the PowerSoil DNA extraction kit (Mobio Laboratories, Carlsbad, CA, United States) according to the kit instructions. The DNA concentration was determined using the NanoDrop 1000 (Thermo Scientific, Waltham, EUA). Then, total DNA was subjected to polymerase chain reaction (PCR) (Majorbio Bio-pharm Technology Co., Ltd, Shanghai, China) prior to sequencing PCR fragments using an Illumina Miseq PE300 platform (Illumina, San Diego, CA, United States). Specifically, total DNA was used as PCR templates, and 16S amplicon libraries were generated using the PCR primers 799F (5′-AACMGGATTAGATACCCKG-3′), 1193R (5′-ACGTCATCCCCACCTTCC-3′), and 1392R (5′-ACGGGCGGTGTGTRC-3′), which span approximately 400 bp of the V5–V7 hypervariable regions of the prokaryotic 16S rDNA gene (Bulgarelli et al., 2012, 2015; Lundberg et al., 2012).

The QIIME2 software platform was used for bioinformatics analysis of raw data derived from the Illumina Miseq platform (Bolyen et al., 2019). Specifically, q2-demux and q2-cutadapt trim-pairs were used to remove barcodes and linkers (Martin, 2011) prior to merging paired-end reads in vsearch (Rognes et al., 2016). The quality-filter and deblur plugins in QIIME2 were used to carry out quality control (Quality Score > 25) and denoising, respectively (Amir et al., 2017). OTUs were clustered based on a 97% similarity threshold determined by the q2-feature-classifier using the SILIVA database (Yilmaz et al., 2014). The diversity plugin of QIIME2 was used to calculate the alpha diversity, weight-unifrac distance matrix, and Bray–Curtis distance matrix. The Vegan package (version: 2.5.6) in R (version: 4.0) (R core team) was used to perform ANOSIM (analysis of similarity) analysis. The calculation of *R* and *P*-values and the comparison of differences between groups were carried out by permutation testing with permutational anova, with the number of permutations set to 999. Two-sided Welch’s *t*-tests with Benjaminii–Hochberg FDR corrections were conducted in the STAMP software (version: 2.1.3) to identify significant differences between sugarcane associated microbial groups (Parks et al., 2014). The Kruskal--Wallis rank-sum test, with an alpha value of 0.05 and a threshold value of 4.8, was used to test for significant differences between microbial groups using an online LEfSe (linear discriminant analysis effect size) program^1^.

### Phylogenetic Tree Construction

Sequences of 16S rDNA from isolates and similar sequences of known bacteria in GenBank database with NCBI BLAST program were downloaded and used for phylogenetic trees analysis in MEGA 7.0 (Kumar et al., 2016). ClustalX^2^ was used for multiple alignments, and the neighbor-joining method in MEGA 7.0 was employed for genetic tree construction (Saitou and Nei, 1987) with 1000 replicates of bootstrap testing (Felsenstein, 1985). Finally, phylogenetic trees were visualized in iTol V4^3^ (Letunic and Bork, 2019) or GraPhlAn (Asnicar et al., 2015).

### Sugarcane Root-Associated Microbe Isolation and Identification

Roots of sugarcane plants grown in the pot cultures described above were used for root-associated microbe isolation. After harvesting sugarcane plants, root samples were collected after first shaking off loosely attached bulk soil, followed by removal of rhizosphere soil firmly attached to the roots through washing with 50 mL of sterile PBS solution for 15 min and centrifugation at 7000 rpm for 8 min (Bulgarelli et al., 2012, 2015). Then, 5 g of root tissues was homogenized in 15 mL of sterile PBS using a mortar and pestle in a laminar flow hood. Plant debris was removed by filtration, and the homogenized solution was considered as the collection of root-associated microbes. Homogenized solutions were diluted 1000-fold with sterile PBS. Finally, 100 μL of diluted microbial suspension was plated on a panel of four microbial isolation culture media and incubated at 28°C (Bai et al., 2015; Wang et al., 2021). The panel of isolation media consisted of two nitrogen free culture media, SNX (Hu et al., 2017) and JNFb (Baldani et al., 1992), and two other culture media containing nitrogen, TSB and M715 (Bai et al., 2015). Single colonies were picked out and streaked to new plates for further purification. Then, single colonies were picked once again from these solid medium plates. All collected isolates from root-associated compartments were subsequently stored in 40% glycerol at –80°C.

To identify potentially nitrogen fixing isolates, PCR assays were employed to identify the N_2_ fixing marker gene (*NifH*) using the primer pair *NifH-F*: AAAGGYGGWATCGGYAARTCCACCAC and *NifH-R*: TTGTTSGCSGCRTACATSGCCATC AT (RöSch et al., 2002). For the determination of potential phosphate solubilizing capacity, isolates were cultured on Pikovskaya agar plates containing Ca_3_(PO_4_)_2_ or phytin as the sole Pi source for 3 days at 28°C. The appearance of a transparent zone around a bacterial colony indicated the phosphate solubilizing capacity. The IAA biosynthesis capacity was measured in a color development assay (Littlewood and Bennett, 2003; Qin et al., 2011). Briefly, isolates were propagated in flasks containing liquid growth media for 3 days at 28°C, followed by centrifugation of 1 mL samples. Cell pellets were washed twice with 1 mL PBS and re-suspended to 10^7^ cells/mL prior to mixing 1 mL of cell suspension with 10 mL of liquid medium containing 100 mol/mL of tryptophan. After incubating for 3 days, 50 μL of supernatant was collected and mixed with 50 μL of Salkowski buffer (4.5 g/L FeCl_3_, 10.8 mol/L H_2_SO_4_). When the solution turns red, it has the ability to synthesize IAA. The colony morphology of SRB-109 was recorded using stereoscopic microscopy.

### Application of Potential Beneficial Microbes in the Fields

For the field application of potentially beneficial microbes. Strains stored at –80°C were melted on ice, streaked on isolation culture medium, and then incubated at 28°C for 5–7 days. Single colonies were then picked off and inoculated in liquid culture medium at 28°C and shaken at 200 rpm until the concentration of bacteria liquid was 1 × 10^8^ CFU/mL. Microbial cells were collected through centrifugation at 7000 rpm for 10 min. After discarding the supernatant, microbial cells were washed three times with low N (200 μM) nutrient solution and finally suspended in low N nutrient solution for later to the concentration of bacteria solution is 5 × 10^7^ CFU/mL.

For sugarcane plant inoculations, seedlings of ROC22 sugarcane were collected at the six-leaf stage from tissue cultures and then immersed in prepared microbial solutions (5 × 10^7^ CFU/mL) for 12 h prior to transplanting into pots filled with sterile substrate and vermiculite in equal proportions. Seedlings were co-cultured with microbial isolates in a growth chamber for 2 weeks, with pots supplied with 20 mL of low N (200 μM) nutrient solution each day (Li et al., 2018). A total of seven pots, with each containing one seedling, were cultivated for each microbial isolate. Control pots contained seedlings that were not inoculated with any microbial isolates. After 2 weeks in a growth chamber, sugarcane seedlings were transplanted to the field with 50 cm spacing between plants for further growth; soil nutrient content and pH of field experimentation are shown in Supplementary Table 1. After cultivation for 120 days in the field, sugarcane plants were harvested for assessment of plant growth promotion.

### Colonization Pattern of SRB-109 on Sugarcane Plants

SRB-109, an isolate from sugarcane roots exhibiting significant growth promotion effects, was selected for further study. To visualize the colonization patterns of SRB-109 on sugarcane plants, SRB-109 was labeled for GUS or red fluorescent protein (RFP) staining with *pMG103-NPTII-GUS* or *pMG103-NPTII-RFP* modified from *pMG103-NPTII-GFP* (Inui et al., 2000) using *Sph* I and *Eco*R I restriction endonuclease sites. Bacteria were labeled by electroporation (Liu S. et al., 2020). Positively transformed bacterial colonies were screened through kanamycin resistance and further identified using GUS staining.

Positively identified GUS labeled bacteria were cultured at 28°C in JNFb liquid medium supplied with 50 μg/mL of kanamycin and shaken at 200 rpm until the concentration of bacteria liquid was 1 × 10^8^ CFU/mL. Bacterial inoculants were prepared through centrifugation at 7000 rpm for 8 min, prior to resuspension in low nitrogen liquid solution to 5 × 10^7^ CFU/mL. Then, 5 mL of prepared inoculum (GUS labeled SRB-109) was added to the liquid nutrient solution of five acclimatized sugarcane plants. After 3 days of co-culturing with GUS labeled microbes, the colonization of SRB-109 throughout various organs of sugarcane plants such as leaves, roots, and whole plants was visualized with GUS staining according to the methods of Li et al. (2015), with the GUS signal being recorded using a stereo microscope (Zeiss Axio Zoom. V16).

### Plant Growth Promoting Genes Identification With Polymerase Chain Reaction

To further confirm the plant growth promoting genes in SRB-109, the genome DNA of SRB-109 was used as a template, the primers specific target to the *nifH* (nitrogenase gene) (RöSch et al., 2002), *ACC* (1-aminocyclopropane-1-carboxylic acid deaminase) (Blaha et al., 2006), and *phoD* (Alkaline phosphatase D) (Sakurai et al., 2008).

## Results

### Structure and Composition of Bacterial Communities Colonizing Root-Associated Compartments of Sugarcane Plants

**FIGURE 1:**
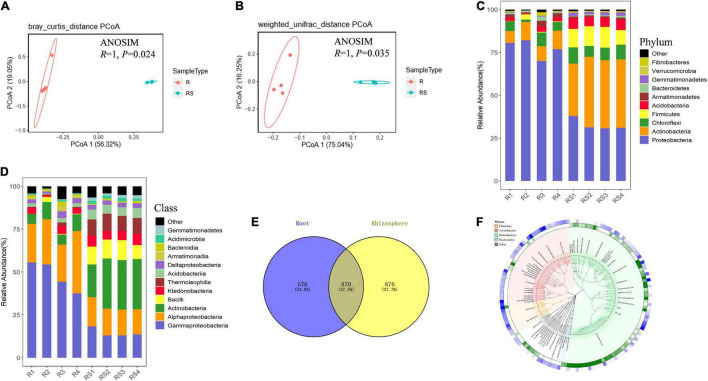
Bacterial communities in the root-associated compartments of sugarcane. Samples were divided into rhizosphere and root samples according to the compartment of origin. Bacterial community composition was determined using 16S rDNA sequencing. Principal coordinates analysis (PCoA) was performed using Bray–Curtis distance **(A)** and weighted-unifrac distance **(B)** based on the OTU table. Analysis of similarity (ANOSIM) was performed based on the OTU tables of R and RS samples to calculate differences. In permutation tests, the number of permutations was 999. The composition of microorganisms was analyzed in the rhizosphere at the phylum level **(C)** and class level **(D)**. R, sugarcane root; RS, sugarcane rhizosphere; OTU, operational taxanomic unit. **(E)** Venn diagram of microbes in the rhizosphere and root compartments of sugarcane. **(F)** Cladogram of top 150 microbes (mean relative abundance) based on taxonomy. OTUs relative abundance of two compartments are shown in the outermost rings of the green and red heat map (root samples as green and rhizosphere samples as blue).

16S rDNA sequencing spanning the V5–V7 regions was employed to investigate the composition of bacterial communities in the rhizospheres and roots of sugarcane plants. A total of 1,149,659 sequences were thusly obtained. After joining paired-end reads, sequences were subjected to quality control and denoising procedures. This yielded 127,810 high-quality 355 bp length reads. Operational taxonomic unit grouping was obtained through clustering of high-quality sequences at the 97% similarity threshold. Rarefaction curves of observed OTU plateaued after 60% sampling (Supplementary Figure 3), suggesting that the sequencing depth for all the samples was enough to cover most of the bacterial in the rhizosphere and root compartments. Bacterial community richness and evenness were significantly higher in the rhizosphere than in the roots, as reflected by the Chao 1 index, the Shannon index, observed numbers of OTU, and the Pielou evenness index (Supplementary Figure 4). This result suggests host involvement in determining root bacterial community members. Further structure analysis based on Bray_Curtis and Weighted unifrac tests all showed that bacterial communities in root compartments can be clearly separated from those in the rhizosphere compartment (Figures 1A,B). An ANOSIM analysis further showed that bacterial communities in the root compartment were significantly different from those in the rhizosphere (Figures 1A,B). All of these results reinforce the notion that roots of sugarcane actively select associated microbial communities.

### Microbes Enriched by Sugarcane

To further investigate which microbes were specifically selected by sugarcane roots. Composition analysis at the phylum taxonomic level showed that *Proteobacteria* was the dominant bacteria in roots, with members of this group accounting for about 77.31% of the relative abundance of total bacteria in sugarcane roots (Figure 1C). In all, *Proteobacteria* (32.68% of rhizosphere bacteria; 77.31% of root bacterial), *Actinobacteria* (37.91%; 9.31%), *Chloroflexi* (7.88%; 5.02%), and *Firmicutes* (10.77%; 0.97%) were the dominant bacteria in the rhizosphere and root compartments of sugarcane plants. At the class taxonomic level, *Gammaproteobacteria* (47.91%; 14.45%) and *Alphaproteobacteri*a (26.79%; 15.63%) were the dominant bacteria in the root and rhizosphere compartments, respectively (Figures 1C,D). In addition, there were 870 OTUs existing in both the rhizosphere and root compartments, while 576 OTU were found exclusively in the root compartment (Figure 1E). The phylogenetic relationship of microbes detected through high throughput sequencing suggests that the sum of the sugarcane associated microbial community originated from a variety of phyla (Figure 1F).

**FIGURE 2:**
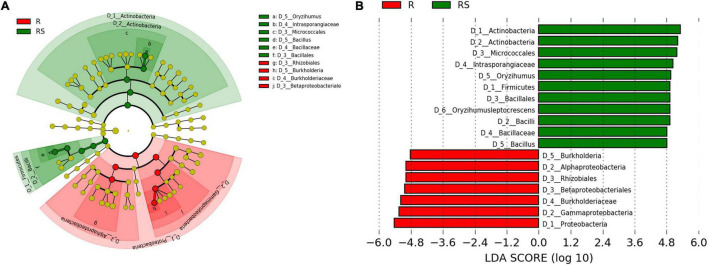
Comparison of bacteria between the rhizosphere and root compartments. **(A)** Phylogenetic dendrogram of biomarkers in the R and RS sugarcane groups. Circles from inside to outside indicate bacterial taxonomic levels from phylum to genus. Yellow dots represent bacteria not significantly varying in abundance among treatments. Biomarker bacteria are colored according to their corresponding class colors on the right. **(B)** LDA scores of biomarker bacteria for each combination of sugarcane sites. LDA scores are shown as horizontal bars for the biomarker bacteria with an LDA score > 4.8 as listed on the left, Kruskal–Wallis rank sum test, *P* < 0.05. LDA, linear discriminant analysis.

To further investigate which microbes were significantly enriched by sugarcane, LEfSe analysis was employed. Results showed that *Gammaproteobacteria*, *Alphaproteobacteria*, *Burkholderiaceae*, *Rhizobials*, and *Burkholderia* were all significantly enriched in the root compartment, while *Actinobacteria*, *Intrasporangiaceae*, *Firmicutes*, and *Bacillus* were enriched biomarkers in the rhizosphere compartment (Figure 2). Meanwhile, STAMP results were similar to LEfSe results (Supplementary Figure 5). Taken together, the observations herein show that *Rhizobials* and *Burkholderia* were significantly enriched in the root compartment and *Bacillus* taxa were mainly enriched in the rhizosphere.

### Isolation and Identification of Beneficial Microbes in Sugarcane

**FIGURE 3:**
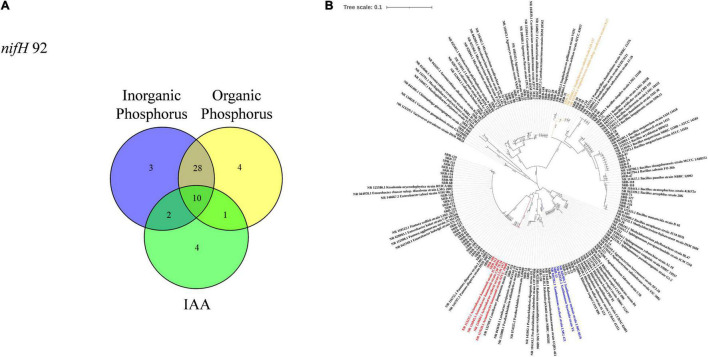
Isolation and identification of microbes from roots of sugarcane. **(A)** Distribution of potentially beneficial functions among isolated root-associated microbes represented in a Venn diagram. **(B)** Phylogenetic tree was constructed using known sequences exhibited similar sequence similarity in GenBank database with NCBI BLAST program. Multiple sequence alignment was done using ClustalX 1.8 software package (http://wwwigbmc.ustrasbg.fr/BioInfo/clustalx), tree was constructed by neighbor-joining method using MEGA 7.0 (Kumar et al., 2016).

To isolate microbes enriched within the root compartment of sugarcane plants, four bacterial culture media were employed, including two nitrogen-free and two of nitrogen-rich media. Isolation and purification procedures yielded a total of 519 isolates from sugarcane roots. These strains were tested for potential nitrogen fixation through PCR amplification of *nifH* genes. Microbes with positive *nifH* gene PCR results were further screened for phosphate-solubilizing capacity and phytohormone IAA production as described in the methods. In these tests, 92 isolates were identified with potential nitrogen fixing capabilities. Among these potential nitrogen fixing members of the sugarcane root flora, 52 returned positive results in one or more of the three other functional assays. Of these, 17 isolates were able to produce IAA, 43 exhibited the capacity of solubilizing inorganic phosphate, and 43 could solubilize organic phosphate. Forty-one potential nitrogen fixing isolates returned positive results in at least two of the IAA production and phosphate solubilizing assays. Ten isolates yielded positive results in all four functional assays (Figure 3A and Table 1). All 92 of the potentially beneficial isolates were subjected to taxonomic assignments and phylogenetic analysis based on 16S rDNA sequences. These results showed that the 92 potentially beneficial strains were *Actinobacteria* (14.13%), *Bacilli* (28.26%), *Alphaproteobacteria* (10.86%), *Betaproteobacteria* (6.52%), *Gammaproteobacteria* (38.06%), and *Sphingobacteriia* (2.17%) (Figure 3B and Supplementary Table 2). The taxonomic grouping of these isolates was mostly consistent with the distribution of all dominant bacterial taxa in the root associated compartments of sugarcane plants (Figure 1F).

**TABLE 1:** 16S rDNA sequence and functional characteristics of nitrogen-fixing bacteria in sugarcane roots.

Strain	Genus	Nitrogenase activity	IAA production	Inorganic phosphate solubilization	Organic solubilization
SRB-1	*Bacillus* sp.				
SRB-4	*Sphingomonas* sp.	+			
SRB-7	*Microbacterium* sp.				
SRB-8	*Bacillus* sp.				
SRB-13	*Xanthomonas* sp.		+	+	
SRB-14	*Sphingomonas* sp.				
SRB-15	*Rhizobium* sp.				
SRB-23	*Rhizobium* sp.				
SRB-26	*Methylobacterium* sp.				
SRB-27	*Curtobacterium* sp.				+
SRB-28	*Staphylococcus* sp.				
SRB-29	*Curtobacterium* sp.		+		
SRB-30	*Staphylococcus* sp.				
SRB-31	*Aeromicrobium* sp.				
SRB-33	*Staphylococcus* sp.	+			+
SRB-35	*Staphylococcus* sp.				
SRB-36	*Curtobacterium* sp.				
SRB-37	*Rhizobium* sp.		+		
SRB-38	*Paraburkholderia* sp.	+			
SRB-40	*Curtobacterium* sp.				
SRB-42	*Leifsonia* sp.				
SRB-43	*Paraburkholderia* sp.				
SRB-45	*Bacillus* sp.				
SRB-47	*Bacillus* sp.				
SRB-48	*Bacillus* sp.			+	
SRB-49	*Cupriavidus* sp.		+		+
SRB-51	*Paraburkholderia* sp.				
SRB-52	*Microbacterium* sp.				
SRB-55	*Agromyces* sp.				
SRB-56	*Microbacterium* sp.				
SRB-58	*Bacillus* sp.				
SRB-59	*Bacillus* sp.			+	+
SRB-63	*Enterobacter* sp.			+	
SRB-64	*Paenibacillus* sp.				
SRB-66	*Paenibacillus* sp.			+	+
SRB-67	*Bacillus* sp.				
SRB-68	*Enterobacter* sp.		+	+	+
SRB-72	*Enterobacter* sp.		+	+	+
SRB-73	*Bacillus* sp.				
SRB-74	*Enterobacter* sp.			+	+
SRB-75	*Brevibacterium* sp.				
SRB-77	*Enterobacter* sp.		+	+	+
SRB-78	*Paenibacillus* sp.		+	+	+
SRB-79	*Enterobacter* sp.		+	+	+
SRB-81	*Enterobacter* sp.		+	+	+
SRB-82	*Rhizobium* sp.		+	+	+
SRB-83	*Bacillus* sp.		+		
SRB-84	*Enterobacter* sp.		+	+	+
SRB-87	*Bacillus* sp.				
SRB-89	*Pantoea* sp.				
SRB-90	*Microbacterium* sp.				+
SRB-91	*Variovorax* sp.				
SRB-92	*Enterobacter* sp.		+	+	+
SRB-96	*Enterobacter* sp.		+	+	+
SRB-97	*Enterobacter* sp.			+	+
SRB-98	*Enterobacter* sp.			+	+
SRB-99	*Enterobacter* sp.			+	+
SRB-102	*Acinetobacter* sp.			+	+
SRB-103	*Enterobacter* sp.			+	+
SRB-105	*Bacillus* sp.			+	+
SRB-106	*Pantoea* sp.			+	+
SRB-107	*Enterobacter* sp.			+	
SRB-109	*Acinetobacter* sp.	+		+	+
SRB-110	*Bacillus* sp.				
SRB-111	*Bacillus* sp.				
SRB-112	*Acinetobacter* sp.	+		+	+
SRB-113	*Enterobacter* sp.			+	+
SRB-115	*Enterobacter* sp.			+	+
SRB-117	*Microbacterium* sp.			+	+
SRB-118	*Bacillus* sp.				
SRB-120	*Pantoea* sp.				
SRB-121	*Pantoea* sp.			+	+
SRB-122	*Enterobacter* sp.			+	+
SRB-124	*Enterobacter* sp.			+	+
SRB-125	*Enterobacter* sp.			+	+
SRB-126	*Bacillus* sp.				
SRB-127	*Bacillus* sp.				
SRB-128	*Enterobacter* sp.			+	+
SRB-129	*Enterobacter* sp.			+	+
SRB-130	*Ralstonia* sp.			+	+
SRB-131	*Enterobacter* sp.			+	+
SRB-132	*Sphingomonas* sp.				
SRB-134	*Paenibacillus* sp.				
SRB-140	*Pantoea* sp.			+	+
SRB-141	*Enterobacter* sp.			+	+
SRB-142	*Enterobacter* sp.			+	+
SRB-144	*Shinella* sp.	+			+
SRB-145	*Mucilaginibacter* sp.			+	+
SRB-149	*Curtobacterium* sp.			+	+
SRB-153	*Luteibacter* sp.		+	+	
SRB-155	*Agrobacterium* sp.		+		
SRB-156	*Chitinophaga* sp.				

**FIGURE 4:**
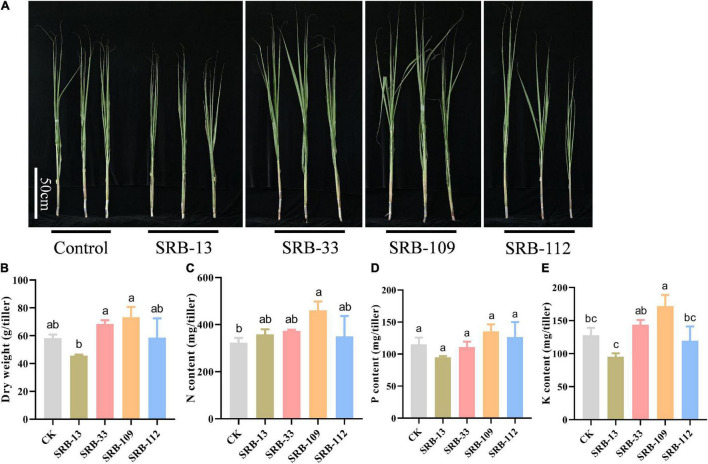
Results of applying potentially beneficial microbes in a field experiment. **(A)** Growth performance of single stem of sugarcane plants in the field trial 120 days after inoculation with microbial isolates, bar = 50 cm. **(B)** Biomass of single stem of sugarcane under differnet treatment conditions. Total nitrogen content **(C)**, phosphate content **(D)**, and potassium content **(E)** of sugarcane plants under different microbial applications under field conditions. Different letters indicate significant differences among different treatments in Duncan’s multiple range comparison test.

After conducting greenhouse assays of plant-microbe interactions, four isolates (SRB-13, SRB-33, SRB-109, SRB-112) displaying obvious growth promotion capacities were selected as candidate strains for further functional validation in a field experiment (Supplementary Figure 6). Phylogenetic analysis showed that the four selected strains were a *Xanthomonas* sp. (SRB-13), a *Staphylococcus* sp. (SRB-33), and 2 *Acinetobacter* sp. (SRB-109 and SRB-112) (Figure 3B). Relative to control treated plots, isolate SRB-109 significantly increased plant height by 27.6%, the leaf SPAD value by 11.7%, and the number of tillers by 6 ± 1. The only other isolate to produce significant improvements in sugarcane traits in these trials was SRB-33, which significantly increased plant height by 27.6% and the number of tillers by 7 ± 1 (Supplementary Figures 8A–C). No isolate significantly impacted dry weight of individual tillers relative to control tillers (Figures 4A,B). Nutrient acquisition relative to control plants was significantly enhanced with SRB-109 treatment by 35.7% for nitrogen and by 28.4% for potassium, but not for phosphorus with this isolate, nor for any nutrients with any of the other three tested isolates (Figures 4C–E). Taken together, these results suggest that SRB-109 may be the most promising isolate identified for promoting sugarcane growth and nutrient acquisition.

### Colonization Patterns of SRB-109 on Sugarcane Plants

**FIGURE 5:**
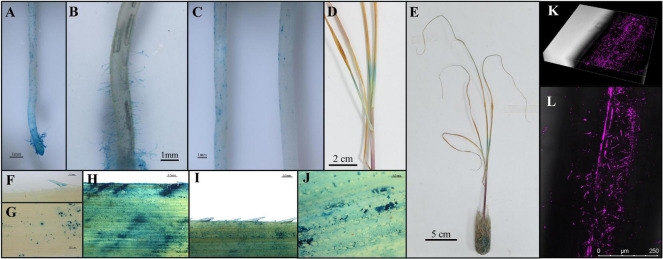
Colonization pattern analysis of SRB-109 on sugarcane. GUS labeled SRB-109 was inoculated and co-cultured with sugarcane for 3 days in the growth chamber. Then, different organs of sugarcane plants were harvested for GUS staining. The blue color indicates the colonization of SRB-109 on different organs, including roots **(A–C)** and leaves **(D–G)**, along with GUS signals detected near inoculation wounds on leaves **(H–J)**. Results of colonization patterns of SRB-109 (RFP labeled) on the roots of sugarcane visualized with confocal microscopy **(K,L)**.

Since SRB-109 exhibited the most promising impacts on sugarcane growth-promotion capacity among tested isolates, this isolate was, therefore, selected for further study of interactions with sugarcane plants. Colony morphology showed SRB-109 with light yellow color, shiny smooth surface, and clear colony edge (Supplementary Figure 7A). And the plant growth promoting genes such as *phoD*, *ACC* deaminase and *nifH* (Supplementary Figure 7B). Taxonomic identification using 16S rDNA showed that SRB-109 is an *Acinetobacter* sp. (Figure 3B). This isolate not only possesses a *NifH* gene but also exhibits the capacity to solubilize both inorganic and organic forms of normally insoluble phosphate (Table 1). Further investigation of SRB-109 colonization patterns using the labeled GUS and RFP reporter gene (Supplementary Figure 9) showed that 3 days after SRB-109/*pMG103-NPTII-GUS* inoculation, an obvious blue signal was detectable on the different tissues of roots, including root tips and root hairs, suggesting that SRB-109 could quickly colonize roots of sugarcane (Figures 5A–C), which was further confirmed by RFP labeled SRB-109 and visualized with confocal microscopy (Figures 5K,L). At the same time, the blue signal of GUS staining was also observed in sugarcane leaves, which suggests that SRB-109 may also colonize the aboveground parts of sugarcane plants (Figures 5D–J).

## Discussion

Plant-associated microbiota are essential for proper host plant growth and health (Niu et al., 2017). During the long history of co-evolution between host plants and their particular suites of microbiota, host plants have evolved several strategies to recruit specific microbes from surrounding soil environments (Mendes et al., 2011; Prashar et al., 2014). These strategies include changing rhizosphere soil pH and texture, production of antibiotic and Quorum-sensing mimicry substances, and specific signaling based on the composition of individual root exudates and cell debris (Marschner et al., 1986; Dennis et al., 2010). Overall, plant associated microbial community composition and activities result from the complex interactions between soil types, geographic conditions, nutrient status, and genotypes of host plants and the available microbial pool (Zhang et al., 2019).

In this study, *Proteobacteria*, *Chloroflexi*, *Actinobacteria*, and *Firmicutes* were the dominant bacteria in the rhizosphere of sugarcane plants at the phylum taxonomic level (Figure 1C). Microbes in these phyla have previously been reported as the core microbiota of sugarcane plants along with *Bacteroidetes*, *Spirochaetae*, and *Verrucomicrobia* (Yeoh et al., 2016). The latter three listed taxa from the previous study were only found in low abundance in this study (Figure 1C), which may due to variation in the soil types and sugarcane genotypes between that published work and this report (Yeoh et al., 2016; Zhang et al., 2019; Liu W. et al., 2020). Consistent with previous studies, *Actinobacteria* was much more abundant than other taxa detected in the root compartments of sugarcane (Figure 2; Dong et al., 2018; Gao et al., 2019). At the genus level, *Leptothrix* sp. were the most abundant microbe in sugarcane roots, though its functions there remain mysterious due to a lack of reports on its functions or interactions with plants. In addition, *Burkholderia* sp. and *Bradyrhizobium* sp. also exhibited higher relative abundances in the sugarcane roots observed herein, with LEfSe analysis showing that these bacteria were significantly enriched in the roots relatvive to the rhizosphere. *Burkholderia* sp. and *Bradyrhizobium* sp. have also been detected from sugarcane plants cultivated in Yunnan Province, China (Dong et al., 2018), though they have been rare or absent in sugarcane grown in Brazil and Australia (de Souza et al., 2016; Yeoh et al., 2016; Gao et al., 2019; Liu W. et al., 2020). Previous work has also concluded that *Burkholderia* sp. and *Bradyrhizobium* sp. may be common plant growth promoting bacteria (Bernabeu et al., 2015; Cagide et al., 2018). However, these two genera were rarely isoalted in this study (Figure 3B and Supplementary Figure 2), which might be due to the composition of the culture medium used to isolate microbes (Bonnet et al., 2019). These results suggest that geographic location, culture conditions, and genotypic variation among of sugarcane plants coordinately regulate the composition of microbiota associated with the sugarcane hosts.

Pure culturing of microbes is important for accurate determination of specific microbial functions (Singh et al., 2019). Over the course of recent decades, with the development of high throughput next-generation sequencing, amplicon sequencing and metagenomics have been widely applied in microbial ecology investigations, with large numbers of microbes being discovered and assigned predicted functions (Giovannoni et al., 1990). To fully assess these predictions requires studying these microbes in controlled settings, typically in isolated cultures. Soil microbes have been historically perceived as largely unculturable (Woese et al., 1990). However, Bai et al. (2015) have demonstrated that 52–65% of *Arabidopsis* associated microbes may be cultured using proper culture media. More recent investigation suggests that up to 97.3% of microbes can be cultured, though most of them have not been investigated (Yang and Jia, 2021). Finally, in sugarcane, 56.1–64.5% of associated microbes may be isolated using broad spectrum microbial media (Armanhi et al., 2017). Therefore, culturing might not be as limiting to functionally characterizing microbial communities as previously perceived.

In this study, considering the importance of associated nitrogen fixation for sugarcane growth (Bai et al., 2015; Hu et al., 2017), two nitrogen-free and two nitrogen-rich media were used to culture and isolate microbes from root-associated compartments. In addition, nitrogen-fixing bacteria associated with sugarcane might also promote sugarcane growth even after the loss of nitrogen fixing capacity, which suggests that associated microbes could also promote plant growth independent of nitrogen fixation (Sevilla et al., 2001). Therefore, after the identification of the nitrogen fixation marker gene *NifH* (Gaby et al., 2018), microbes isolated in this study were also subjected to assays for other potentially beneficial functions relevant to the low phosphorus bioavailability acid soil regions of sugarcane production in South China (Kochian, 2012; Figure 3A). Since not all potentially beneficial microbes actually promote host plant growth, and given the complex interactions between microbes and host plants (Finkel et al., 2017; Wang et al., 2021), isolated microbe–plant interaction assays were necessary in this study to ultimately verify the potentially beneficial functions of isolated microbes. After co-culturing sugarcane seedlings with different selected microbes in greenhouse testing, four isolates were further observed under field conditions. The results of this field trial suggest that SRB-109 exhibited better plant growth-promotion than the other isolates identified in this study (Figure 4). Differences in the performance of the other isolates between greenhouse testing and the field trial might be due to the complex and dynamic field conditions (Finkel et al., 2017). Nevertheless, SRB-109, an apparent *Acinetobacter* sp. (Supplementary Figure 8), significantly promoted sugarcane growth in both greenhouse and field conditions. Laboratory assays suggested that SRB-109 might assist host plants in nutrient acquisition through solubilizing phosphate, as well as through fixing nitrogen (Table 1). However, whether the nitrogen fixing capacity of SRB-109 higher or lower than the previously identified sugarcane associative nitrogen fixing bacteria, such as *Azospirillum* spp. *Herbaspirillum* spp., *Gluconacetobacter* spp., *Enterobacter* spp., and *Burkholderia* spp., it will need further invesstigation and comparision of their nitrogen fixing capacity under the same treament conditions. Previous studies also suggest that *Acinetobacter* sp. may promote host plant growth through phytohormone production along with solubilizing phosphate (Kang et al., 2009; Rokhbakhsh-Zamin et al., 2011; Ishizawa et al., 2020). Consistently, in our study, the application of SRB-109 significanly enhanced the acquisition of nitrogen and potassium (Figure 4), which might be due to the functions of nitrogen fixation (Table 1). In addition, although phosphate solubilizing capacity in SRB-109 was significant in the lab, application of this isolate in the field did not significantly enhance phosphorus acquisition (Figure 4D). Further observation with GUS staining to determine which tissues SRB-109 colonizes suggests that SRB-109 can colonize sugarcane roots and root hairs, as well as, aboveground compartments. These observations indicate that SRB-109 establishes an intimate relationship with sugarcane that benefits the plants through gains in nutirent acquisition capabilities (Figure 5).

## Conclusion

In this study, we used tissue culture of sugarcane seedlings and 16S rDNA sequencing of associated microbiota to systematically decipher the structure and composition of bacterial communities recruited and enriched from soils by sugarcane roots. Isolation of root-associated microbes and screening of the potentially beneficial members allowed for further evaluation of these isolates for the promotion of sugarcane growth in both greenhouse and field experiments. This led to the identification of SRB-109 as a rapid colonizer of both sugarcane roots and shoots that may significantly increase tillering and nitrogen acquisition by sugarcane plants. In this study, we outlined a strategy for functionally studying potentially beneficial plant-associated microbes, and demonstrated effects under field conditions. Building on these results might lead to applications of beneficial plant-associated microbes to decrease fertilizer use and promote the development of sustainable agriculture.

## Data Availability Statement

The 16s rDNA sequencing files for all samples used in this study have been deposited in the public database of the National Center for Biotechnology Information (NCBI) under project number BioProject ID: PRJNA748846.

## Author Contributions

YZ and HL designed the experiments and managed the projects. ML, RL, YL, CW, WM, LZ, KZ, and XF performed the experiments. XL, YS, and GH provided the tissue culture seedling and suggestion in the experiment and manuscript revision. YZ and ML performed the data analysis. YZ, HL, and ML wrote the manuscript. All authors contributed to the article and approved the submitted version.

## Conflict of Interest

The authors declare that the research was conducted in the absence of any commercial or financial relationships that could be construed as a potential conflict of interest.

## Publisher’s Note

All claims expressed in this article are solely those of the authors and do not necessarily represent those of their affiliated organizations, or those of the publisher, the editors and the reviewers. Any product that may be evaluated in this article, or claim that may be made by its manufacturer, is not guaranteed or endorsed by the publisher.

## Footnotes

1
http://huttenhower.sph.harvard.edu/galaxy/root/index

2
http://www.clustal.org/

3
https://itol.embl.de/
