# Supplementary material for: Functional Investigation of Plant Growth Promoting Rhizobacterial Communities in Sugarcane
Source: Front Microbiol. 2022 Jan 4;12:783925. doi: 10.3389/fmicb.2021.783925 (PMC8763851; doi:10.3389/fmicb.2021.783925)
Supplement: Supplementary file 1 [file Data_Sheet_1.docx]

***Supplementary Figures***

**
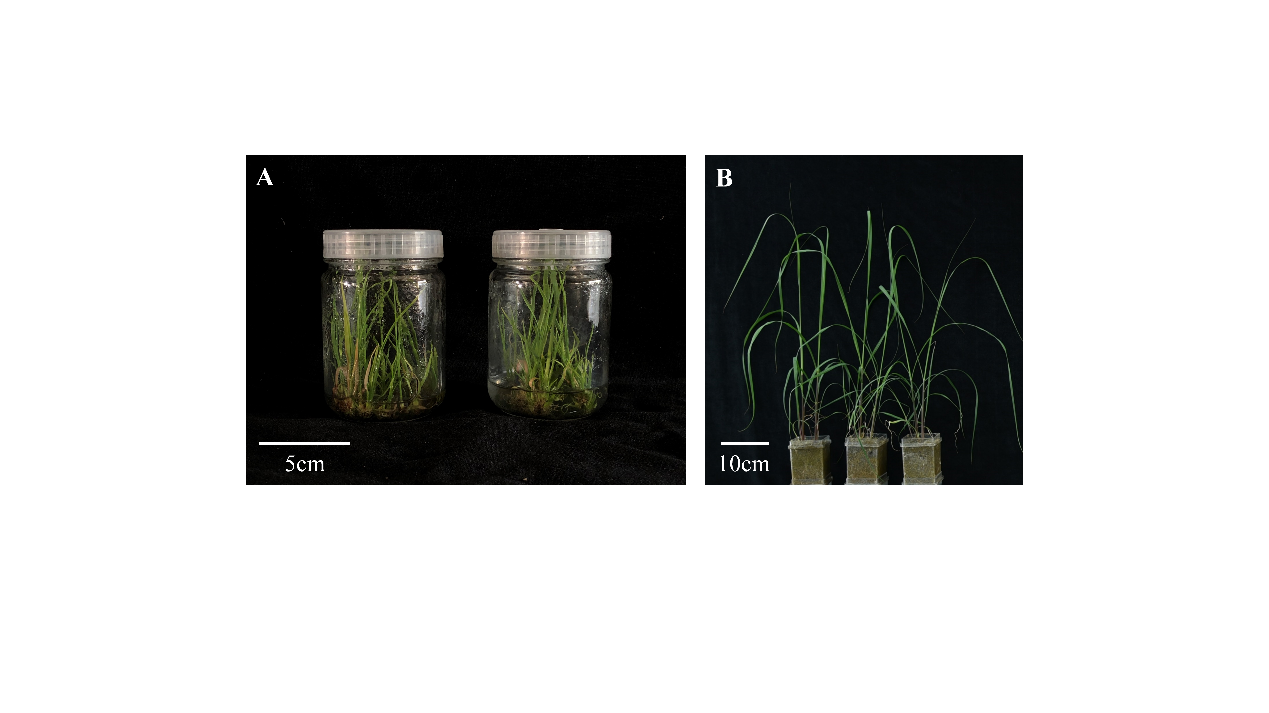
**

**Supplemental Figure 1 Pictures of tissue cultured seedlings and pot cultures of sugarcane plants**

**A** Pictures of tissue cultured sugarcane seedlings at the rooting medium stage in tissue culture bottles. Bar = 5 cm. **B** Picture of sugarcane plants grown in pots during root-associated microbial enrichment experiments conducted in the growth chamber. Bar = 10 cm.


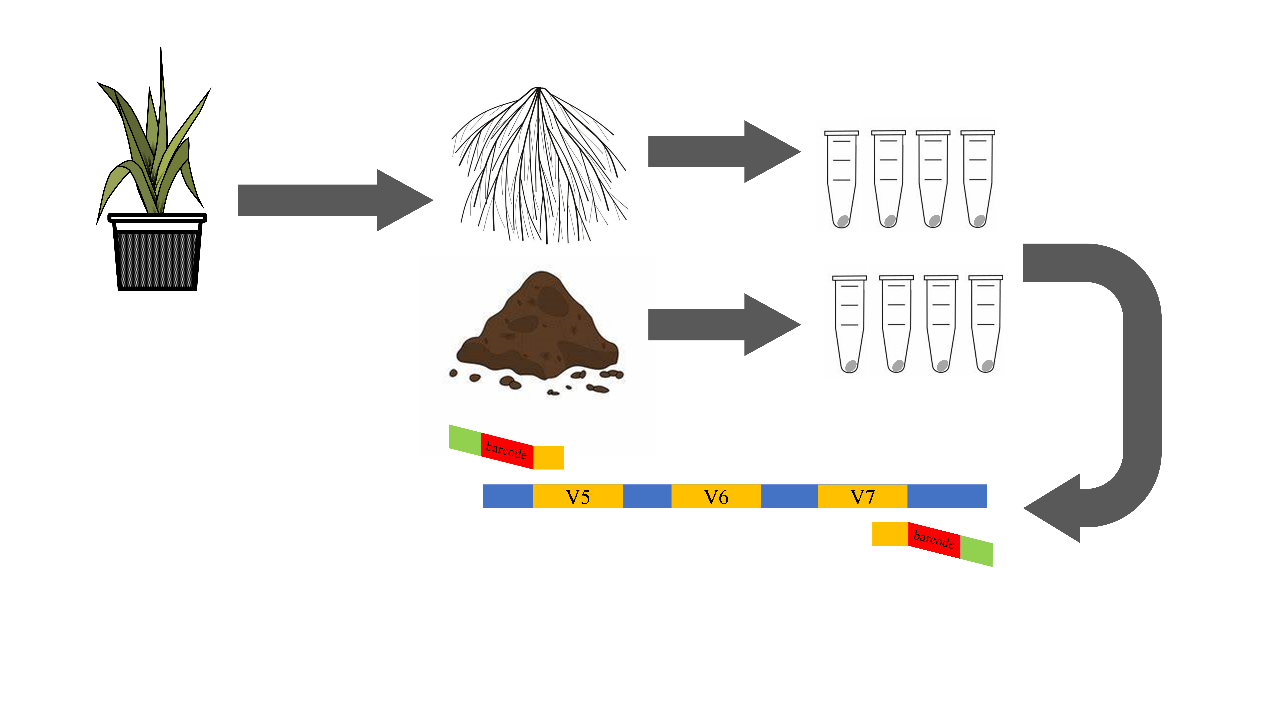


**Supplemental Figure 2 Diagram of root-associated microbial isolation and sequencing**

Sugarcane plants were cultured in pots with soil collected from sugarcane experimental field soil. Rhizosphere soils and root samples were subjected to total DNA extraction and high-throughput sequencing of the V5-V7 regions of 16S rDNA.

**
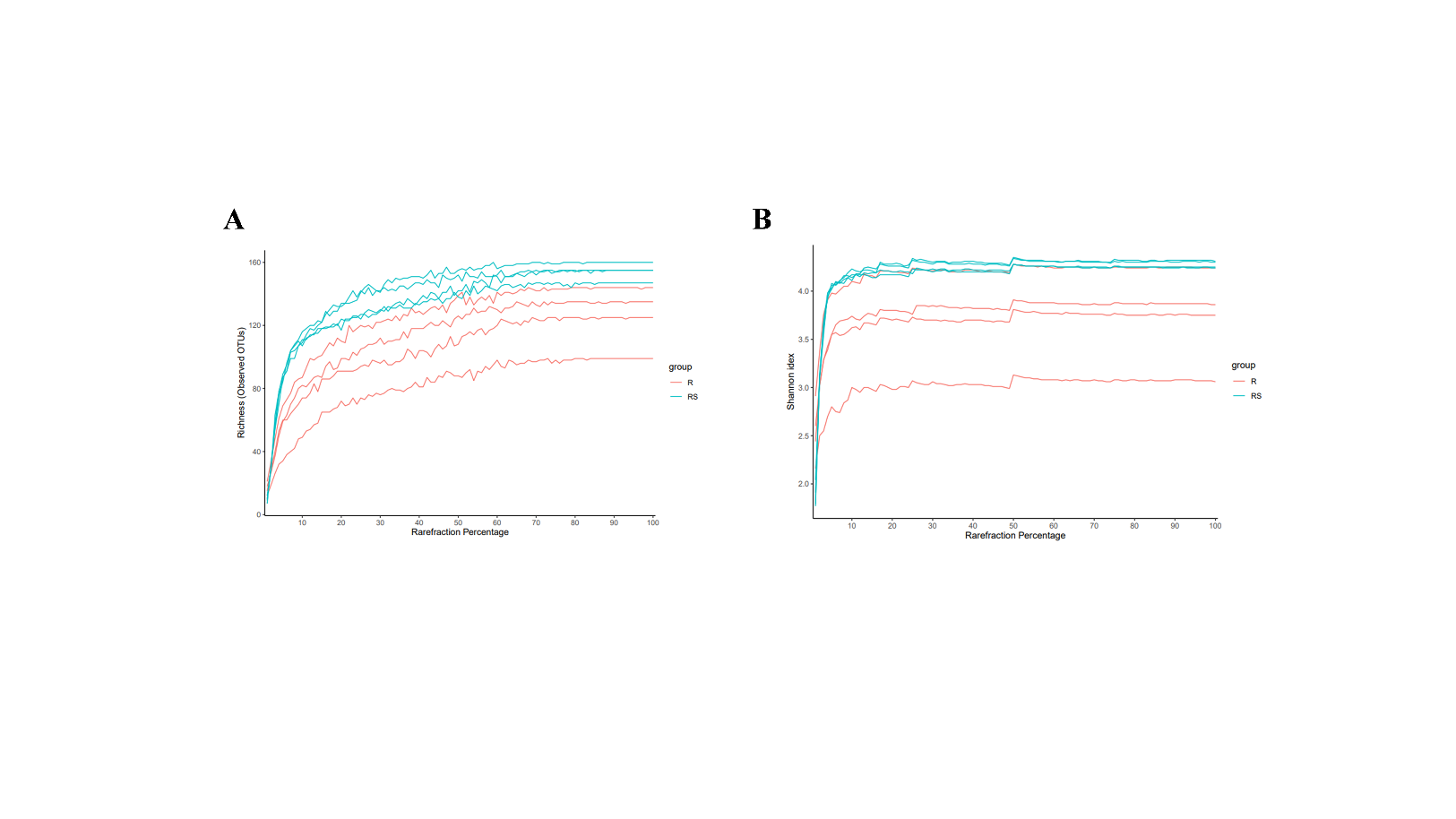
**

**Supplemental Figure 3 Rarefaction curves for sugarcane root and rhizosphere microbe communities**

Rarefaction curves were analyzed by calculating the richness index (**A**) and Shannon index (**B**). Usearch V10 was used for sampling with the sampling step = 1000. The rarefaction curves were generated by ggplot2.


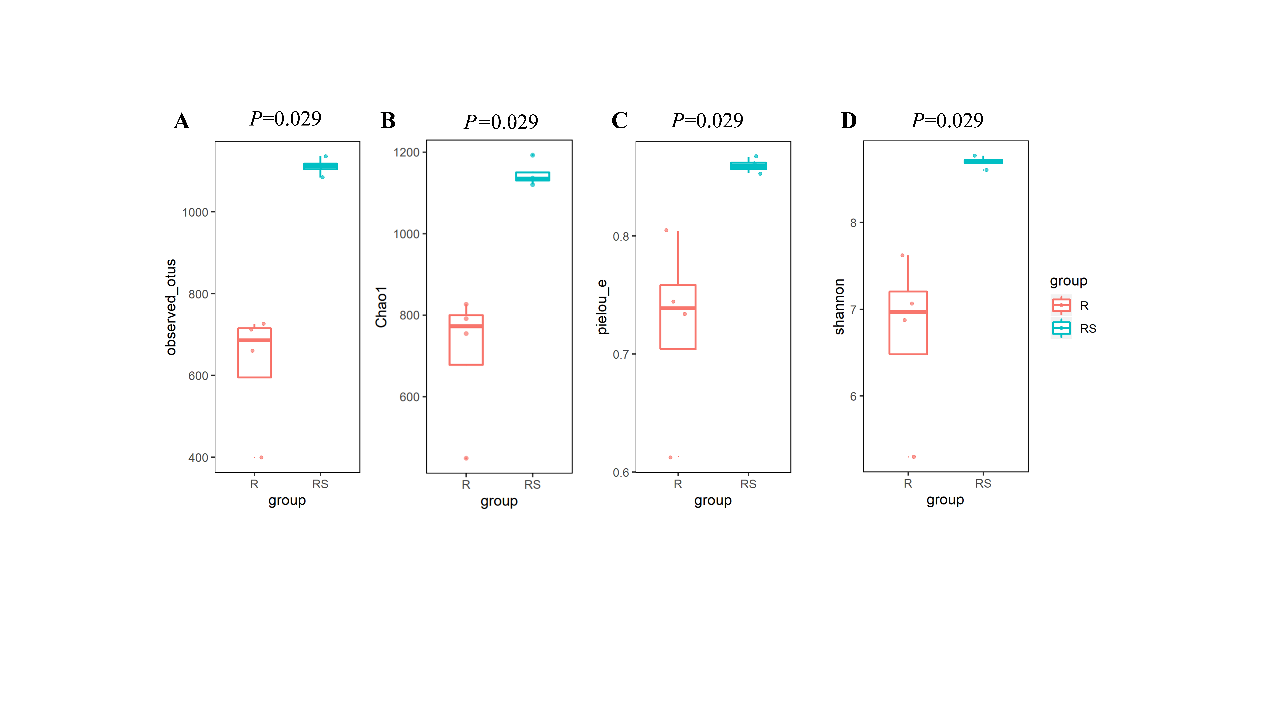


**Supplemental Figure 4 Comparison of bacterial diversity and richness between root and rhizosphere compartments**

Bacterial diversity and richness in the root and rhizosphere compartments of sugarcane were compared using the Observed OTUs (**A**), Chao1 index (**B**), Pielou evenness index (**C**), and Shannon index (**D**). The Mann-Whitney *u*-test was used to test for significant differences.


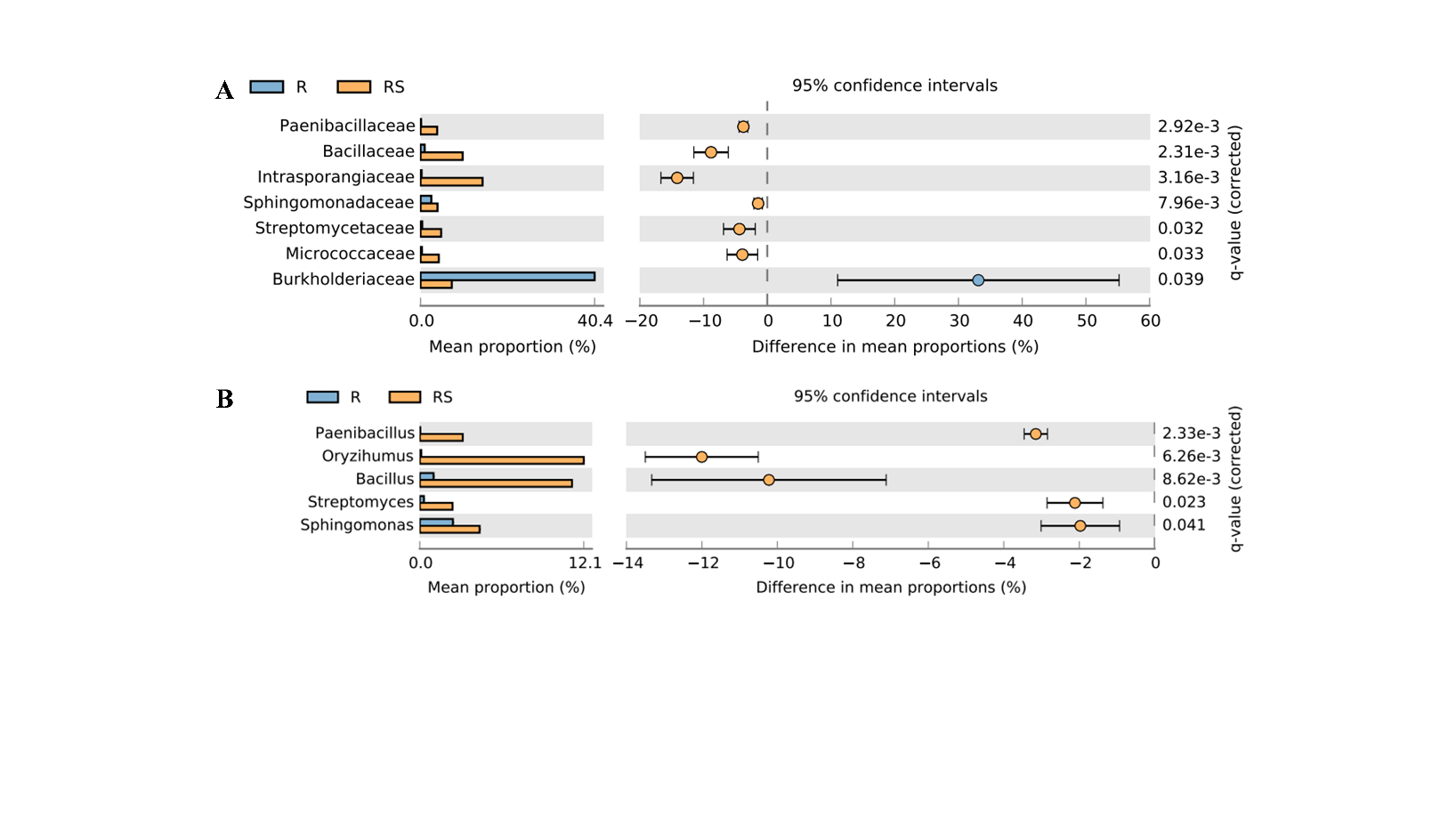


**Supplemental Figure 5 Microbes significantly enriched in the roots of sugarcane**

Microbes significantly enriched in the sugarcane root endo-compartment relative to abundance in the rhizosphere. Bacteria that were strongly selected by sugarcane plants at the family and genus taxonomic levels were analyzed based on relative abundance profiles using STAMP software (version:2.1.3), Corrected *P*-values were calculated in the two-sided Welch’s *t*-test with the Benjamini-Hochberg FDR correction.


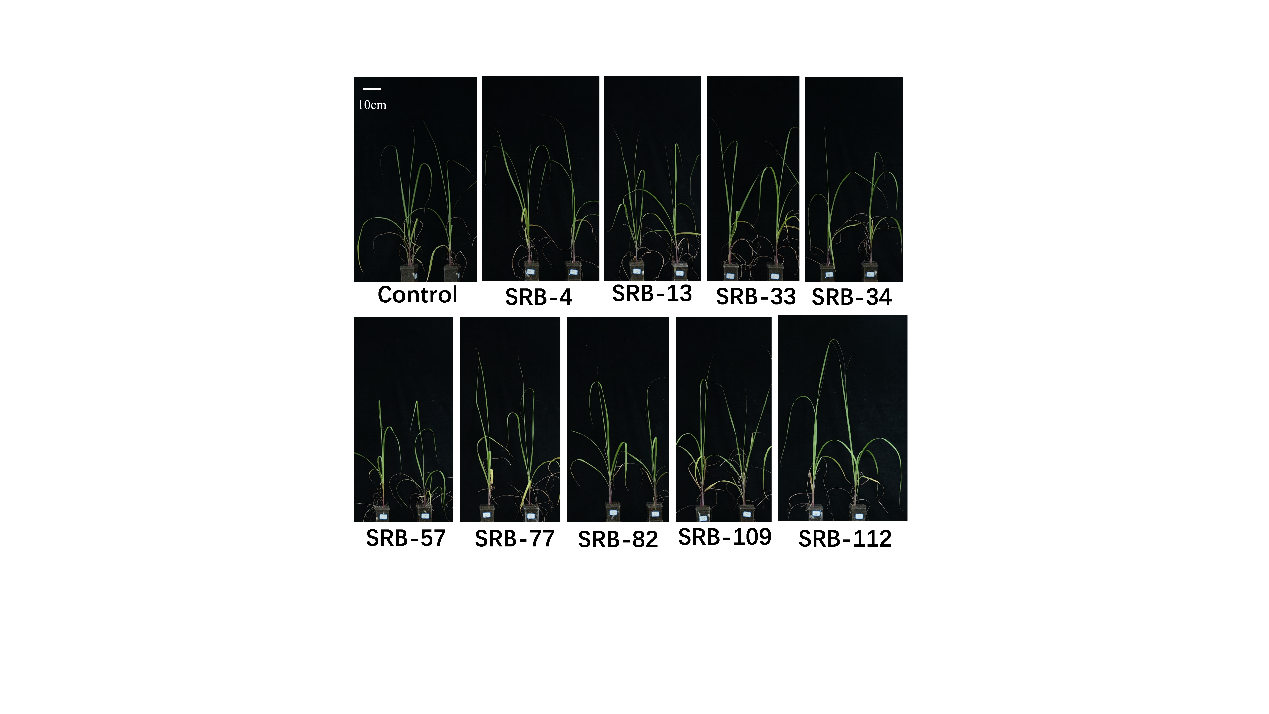


**Supplemental Figure 6 Indoor screening the plant growth-promoting microbes**

Growth performance of sugarcane plant two months co-cultured with selected potential beneficial microbes. Sugarcane seedlings were inoculated and co-cultured with potential bacteria isolated from roots of sugarcane plants.


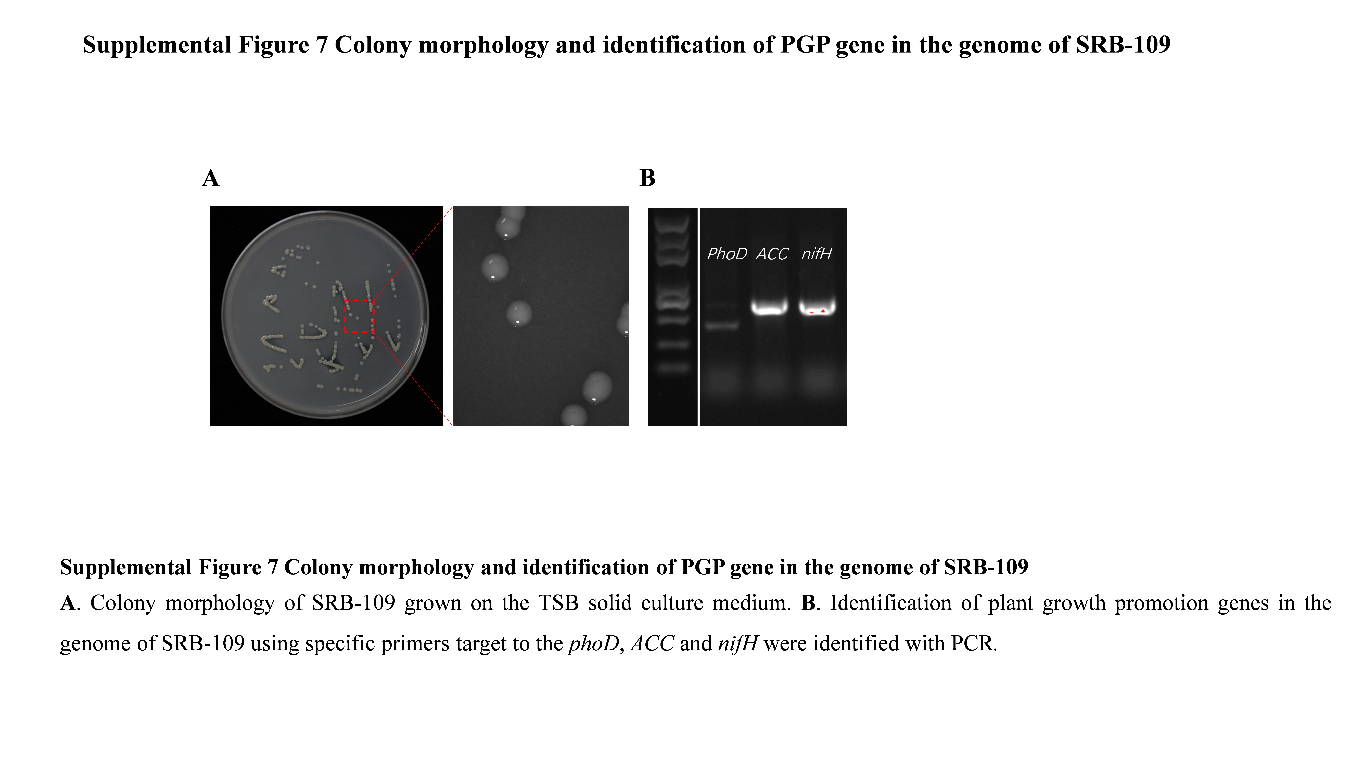


**Supplemental Figure 7 Colony morphology and identification of PGP gene in the genome of SRB-109**

1. Colony morphology of SRB-109 grown on the TSB solid culture medium. **B**. Identification of plant growth promotion genes in the genome of SRB-109 using specific primers target to the *phoD*, *ACC* and *nifH* were identified with PCR*.*


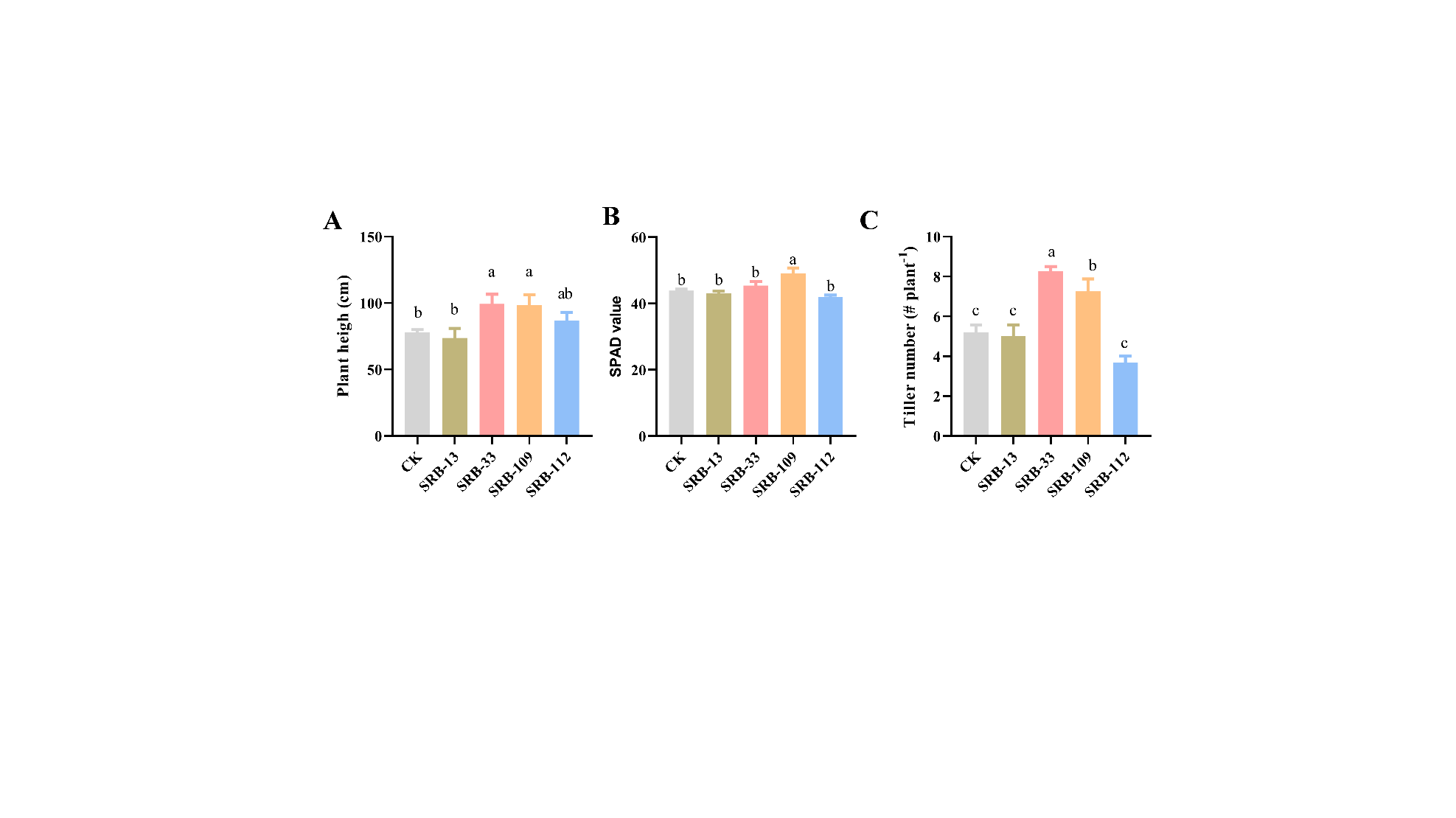


**Supplemental Figure 8 Influence of microbial application to the growth of sugarcane plants in field conditions**

**A** Height of sugarcane plants subjected to different microbial treatments under field conditions. **B** SPAD value of functional sugarcane leaves. **C** Tiller numbers of sugarcane plants inoculated with different microbial isolates. Different letters indicate significant differences among different treatments in Duncan`s multiple range comparisons test.


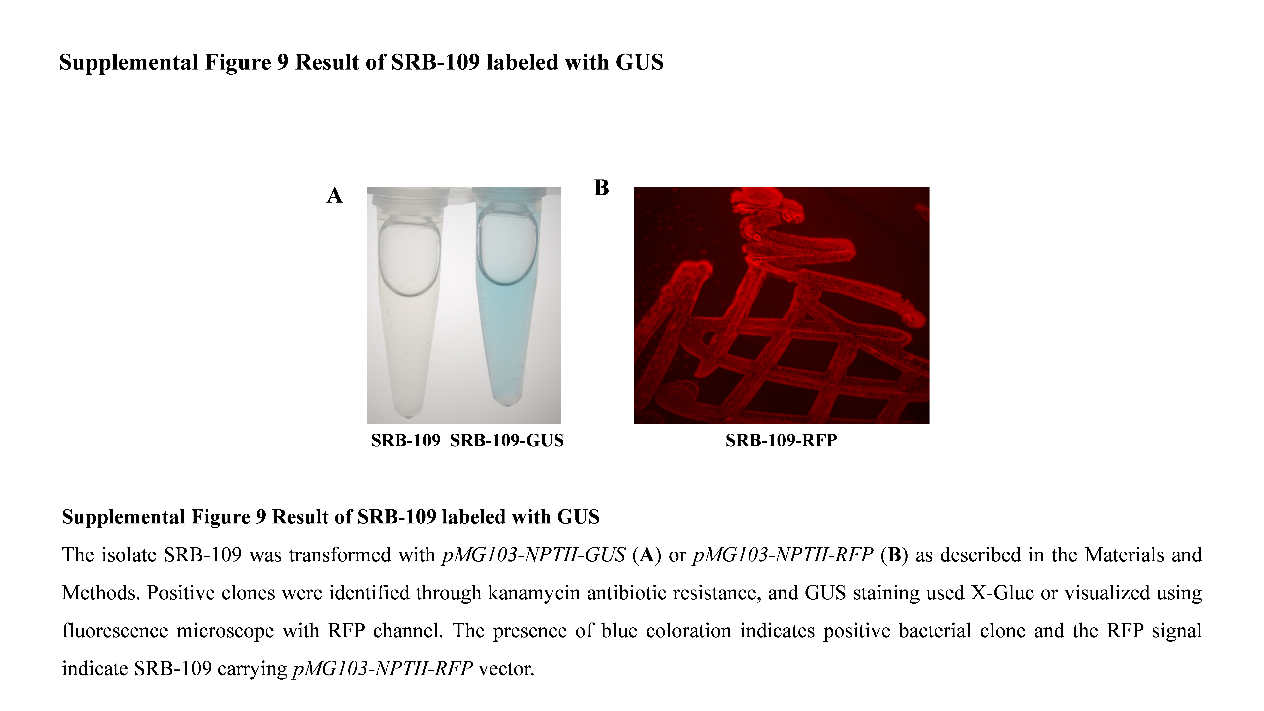


**Supplemental Figure 9 Result of SRB-109 labeled with GUS**

The isolate SRB-109 was transformed with *pMG103-NPTII-GUS* (**A**) or *pMG103-NPTII-RFP* (**B**) as described in the Materials and Methods. Positive clones were identified through kanamycin antibiotic resistance, and GUS staining used X-Gluc or visualized using fluorescence microscope with RFP channel. The presence of blue coloration indicates positive bacterial clone and the RFP signal indicate SRB-109 carrying *pMG103-NPTII-RFP* vector
