# Supplementary material for: Functional Investigation of Plant Growth Promoting Rhizobacterial Communities in Sugarcane
Source: Front Microbiol. 2022 Jan 4;12:783925. doi: 10.3389/fmicb.2021.783925 (PMC8763851; doi:10.3389/fmicb.2021.783925)
Supplement: Supplementary file 2 [file Table_2.DOCX]

**Supplemental Table 1** Soil nutrient content and pH in Yangzhong location of field experimentation

| Field site | pH | Organic matter (g/kg) | Alkali-hydrolyzable  nitrogen (mg/kg) | Available phosphorus  (mg/kg) | Available potassium (mg/kg) |
| --- | --- | --- | --- | --- | --- |
| Yangzhong | 5.42±0.09 | 2.40±0.13 | 100.68±1.28 | 115.95±7.01 | 107.59±7.48 |
